# Orbital Cellutitis and Peri-Zygomatic Cutaneous Fistula After Monolateral Double Zygomatic Implant Placement: Case Report and Narrative Literature Review

**DOI:** 10.3390/dj13080381

**Published:** 2025-08-21

**Authors:** Domenico Sfondrini, Stefano Marelli, Dario De Martis, Andrea Scribante, Giada Beltramini, Luca Autelitano, Lorenzo Preda

**Affiliations:** 1 Department of Maxillo-Facial Surgery, Fondazione IRCCS Policlinico S. Matteo, 27100 Pavia, Italy; d.sfondrini@smatteo.pv.it (D.S.); s.marelli@smatteo.pv.it (S.M.); 2 Section of Dentistry, Department of Clinical, Surgical, Diagnostic and Pediatric Sciences, University of Pavia, 27100 Pavia, Italy; dariodemartis@gmail.com; 3 Maxillo-Facial Surgery and Dental Unit, Fondazione IRCCS Ca’ Granda Ospedale Maggiore Policlinico, 20122 Milano, Italy; giada.beltramini@unimi.it; 4 Maxillo-Facial Surgery Unit, Ospedale ASST San Paolo e Carlo, 20142 Milano, Italy; luca.aute@gmail.com; 5 Department of Clinical, Surgical, Diagnostic and Pediatric Sciences, University of Pavia, 27100 Pavia, Italy; 6 Department of Radiology, Fondazione IRCCS Policlinico San Matteo, University of Pavia, 27100 Pavia, Italy; 7 National Center for Oncological Hadrontherapy (CNAO), 27100 Pavia, Italy

**Keywords:** zygomatic implants, complications of zygomatic implant surgery, cutaneous fistula, zygomatic fistula, inter-implant distance

## Abstract

Background. The use of zygomatic implants (ZIs) provides a highly predictable treatment option for rehabilitation in patients with severe atrophic maxillae. However, these long implants can potentially cause a number of more serious complications than those seen with conventional dental implants. The aim of this study is to report a case of peri-zygomatic cutaneous fistula following placement of monolateral double zygomatic implants and to analyse the available literature on this complication. Methods. The 55-year-old patient was treated with placement of 3 ZIs, two on the left side. Left periorbital swelling with pain appeared 10 days after surgery with progressive worsening of symptoms. After antibiotic treatment, she developed a left cutaneous fistula with purulent discharge. CT showed two ZIs on the left side with the apical portions in close contact with a 1 cm-wide portion of resorbed zygomatic external cortex and a layer of granulation tissue. Results: Due to the limited amount of bone involved by the fixation tip, the left ZIs were removed and the skin fistula repaired. The patient healed without complications but required prosthesis replacement. Conclusions. After conducting a literature review, peri-zygomatic fistulas seem to be more common in patients with two ZIs placed on the same zygoma. In this case, the amount of available zygomatic bone is relatively limited; the bone drill holes can often be too close together and cause overheating, leading to inter-implant bone resorption and infection, with further orbito-zygomatic fistula development. The authors identified the lack of distance between ZI fixtures as one of the main causes of extraoral ZI infection.

## 1. Introduction

Zygomatic implants (ZIs) are considered one of the available techniques for dental rehabilitation in the atrophic maxilla. This concept was introduced in 1998 by Branemark [1] for fixed rehabilitation in oncological patients after maxillectomy. Its use was later extended to healthy patients with severe maxillary atrophy, and the technique has been described as highly predictable with a low failure rate. A recent study (Da Hora Sales et al., 2020) [2] reported an overall survival rate of 96.7%.

ZI treatment, although described in the early literature [3,4,5,6,7,8,9,10,11,12,13,14,15], has not been widely adopted because it is an advanced surgical procedure that can only be performed by experienced clinicians. This procedure requires extensive subperiosteal undermining and knowledge of the orbito-zygomatic anatomy. In fact, long fixation may involve delicate anatomical structures such as the orbit, causing ophthalmological complications [14,15]. In addition, many other complications have been reported in the literature. Sinusitis is the most common, followed by lack of osseointegration, oroantral communication at the fixation level, paraesthesia in the zygomatic area, and cutaneous fistula in the lower eyelid-malar area.

Recently, the introduction of computer-assisted surgery with digital planning and customised guides has made ZIs more feasible and greatly increased their use. Scientific studies on the long-term stability of ZIs are increasing, as are reports of surgical complications. In particular, orbital invasion and periorbital cutaneous fistula are the most troublesome complications to resolve [13,14] and are more difficult to accept for a patient who simply requires prosthetic rehabilitation.

**Figure 1:**
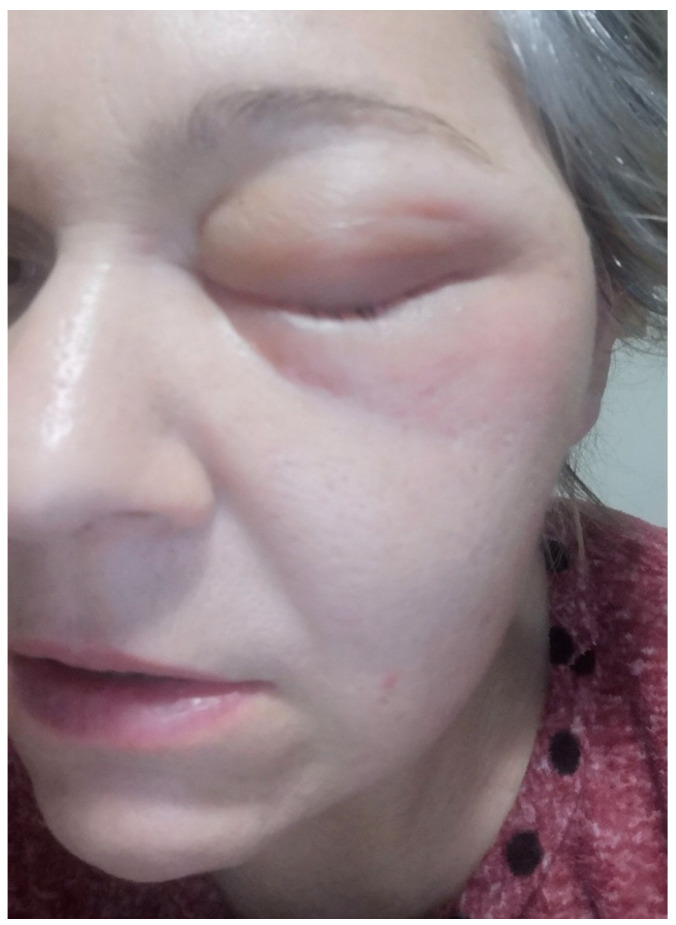
Left periorbital non-coloured painful swelling compared 10 days after unilateral placement of ZIs.

The course of extraoral skin infections follows a standard pattern. Initially, there is a non-coloured, painful swelling in the zygomatic region (Figure 1), followed by an increase in volume and local erythema with abscess formation. If not surgically drained, it will spontaneously develop into a cutaneous fistula. In general, a number of systemic factors increase the risk of infection in implantology: diabetes, microcirculatory pathologies, use of anti-resorptive drugs, and bad habits such as smoking. In general, all these factors reduce blood flow and increase the risk of infection.

The aim of this study is to describe a patient who developed orbital cellulitis and periorbital cutaneous fistula after zygomatic implantation and to analyse all the available literature on this complication. To the best of our knowledge, the authors identified, for the first time, the primary suspected cause as insufficient inter-implant distance between the ZIs. No previous studies have reported this primary cause of periorbital fistula in ipsilateral ZI surgery. Strategies for its prevention and management are provided.

## 2. Case Report

A 55-year-old female patient was referred to the Department of Maxillo-Facial Surgery, Fondazione IRCCS Policlinico S. Matteo, Pavia, Italy, for a cutaneous fistula with purulent discharge in the left zygoma region. The patient did not have diabetes or other microcirculatory disorders, did not smoke, underwent no radiation therapy, and had no other local or systemic problems. She had no history of anti-resorptive medication.

She reported previous placement of 3 ZIs twenty days prior to consultation. Ten days after implantation, redness, swelling, and pain, with inability to open the eye, appeared in the left periorbital area (Figure 1).

**Figure 2:**
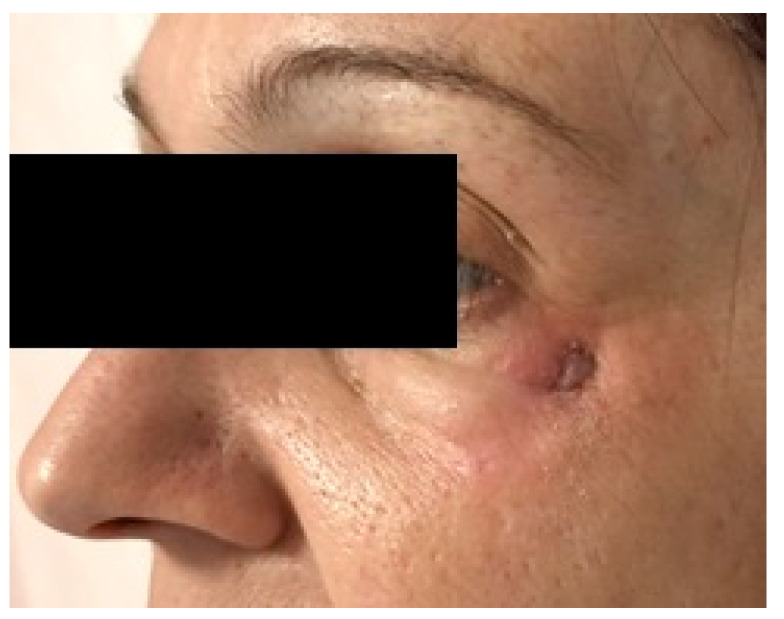
Spontaneous left peri-orbito-zygomatic fistula occurred 20 days after ZIs placement.

With 5 days of antibiotic therapy the symptoms improved and, after 2 weeks, the patient developed a cutaneous fistula in the left zygomatic area with spontaneous drainage of the abscess (Figure 2).

**Figure 3:**
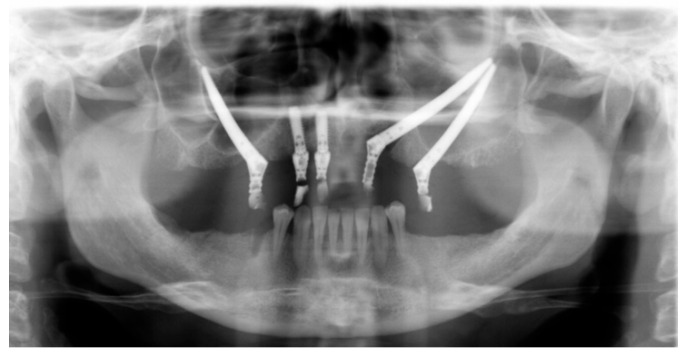
Panoramic radiograph, showing two ZIs on the left side with their apex in close contact without adequate inter-implant distance.

**Figure 4:**
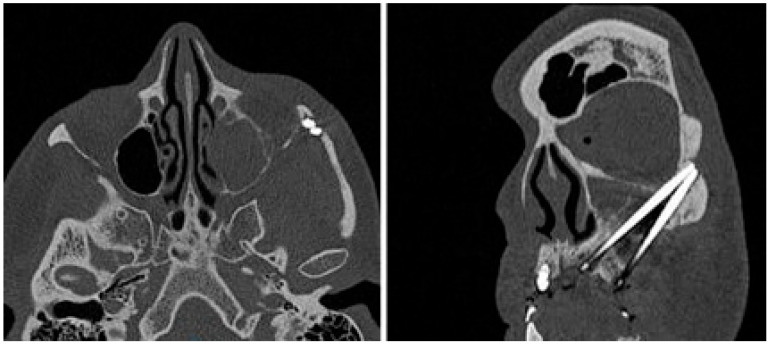
CT axial and coronal view: two ZIs on the left zygoma with apex in contact without adequate inter-implant distance and outer cortical bone resorption.

At the intraoral inspection, after removing the overdenture, no signs of infection were revealed. On the orbito-zygomatic region a skin fistula was present (Figure 2). The orthopantomography showed on the right side one zygomatic implant along with two conventional fixtures in the premaxilla area. A CBCT was performed. On the left side 2 ZIs were inserted with emergence, respectively, in the canine and in the premolar area. The etched apical portions appeared to be almost in contact, with 1 mm distance or less between them. (Figure 3 and Figure 4).

**Figure 5:**
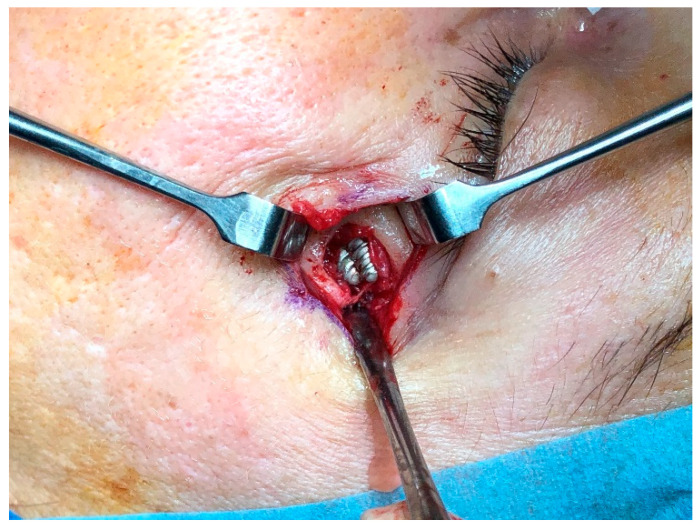
After removing the skin fistula, a 10 mm diameter lack of bone area is shown. The etched parts are in close contact without inter-implant distance.

The implant apex was not palpable under the skin of the zygomatic bone. The patient was operated on under general anaesthesia with intraoral and external approach. The cutaneous fistula was excised and after removing the granulation tissue, 1 cm diameter bone resorption was noted (Figure 5), confirming the CT reports (Figure 4). The Zis apex was in contact, without inter-implant distance (Figure 4).

**Figure 6:**
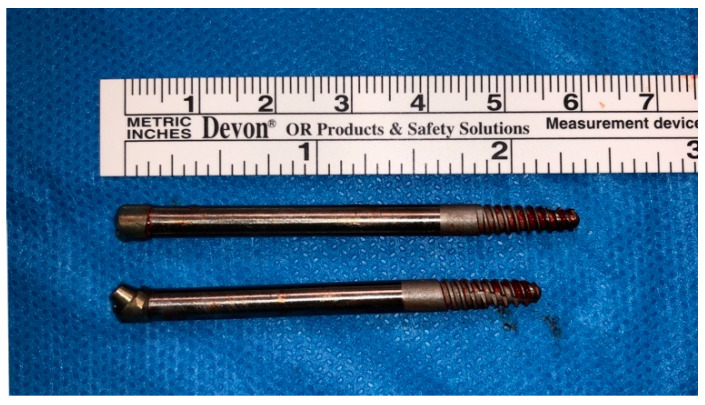
Implants removed with abutment. The zygomatic implants have 3.80 mm diameter and 51–57 mm length. A sandblasted and etched 15 mm apical portion is noted.

**Figure 7:**
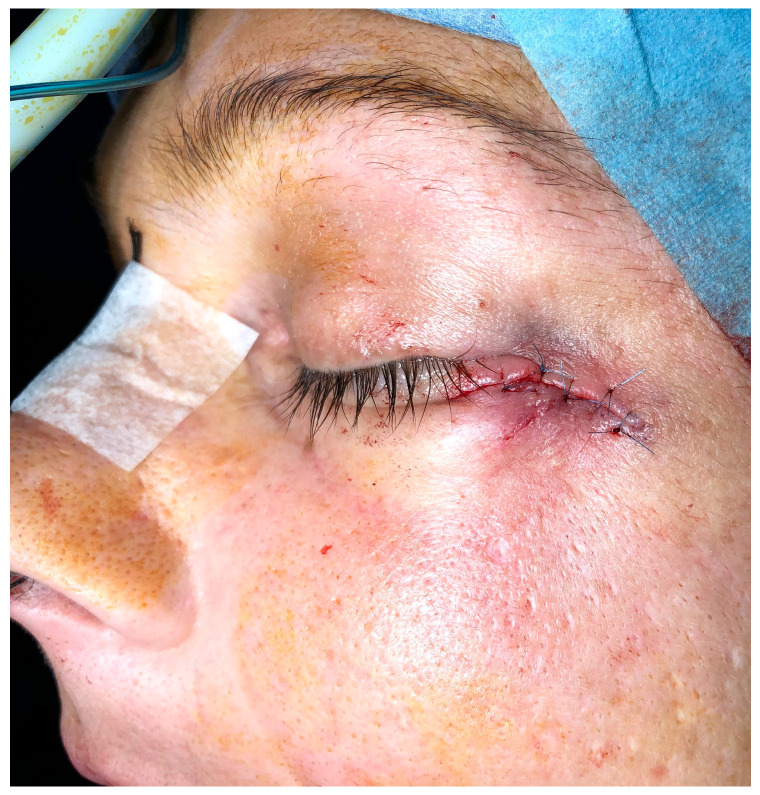
Skin fistula repaired.

As the zygomatic bone around the ZIs was significantly reduced and the mechanical implant stability compromised, the authors preferred to remove it through oral approach (Figure 6). Bone curettage and a fistula repair were performed (Figure 7). The patient progressed well and was discharged on the first postoperative day. After follow-up, she was referred to her dentist for prosthetic rehabilitation.

## 3. Discussion

ZI placement is not without risk and many surgical complications have been reported. Extra-oral complications such as orbital implant penetration and orbito-zygomatic cutaneous fistula are fortunately extremely rare.

All implants affected by cutaneous fistulas require surgical treatment. Conservative treatment with surgical removal of the involved apical part of the implant (apicoectomy) can be effective in some cases [15,16,17,18,19,20,21,22]. In other patients, where apicoectomy has failed to cure the infection or where the mechanical retention of the implants has been compromised, as in the patient presented, total implant removal is required [22].

From the literature review, there are few reports of the development of cutaneous fistulae following ZI placement.

In these studies [15,17,18,19,20,21,22] the authors stated that the probable cause of orbital zygomatic infection and subsequent fistula could be an accumulation of drilling debris (bone, periosteum, and sinus membrane particles) remaining in the more apical part of the surgical site, which is difficult to reach by normal irrigation. These can become infected by bacteria transferred from the mouth to the zygomatic subcutaneous tissue. Poor irrigation in the apical area of the bone hole can even lead to bone necrosis, hypothetically caused by overheating. In addition, thermal damage is more likely to occur in dense bone such as the zygoma than in spongy bone [23]. Over-torquing may also be a potential cause of infection: under-preparation of the zygomatic surgical site increases the insertion torque when placing ZIs and can lead to slowly developing necrosis, possibly taking years to develop a cutaneous fistula [20]. In fact, the timing of presentation can vary considerably; infection can develop weeks or even years after implantation. The timing of the appearance of the complication allows us to deduce the cause. If the complication occurs after many months or years, overtightening is a reasonable explanation; if it occurs after a few weeks or months, the most likely cause is overheating in the apical area of the osteotomy during the drilling procedure. Intuitively, the timing of the development of periorbital infection is also related to the general health of the patient and the local immune status, data that are rarely described in the literature reviewed.

According to other authors, the primary cause of cutaneous fistulae is the protruding tip of the zygomatic implant, which causes chronic irritation and inflammation in the overlying soft tissues, leading to infection and fistulation [19]. According to the authors, this can cause mild swelling and pain in the overlying skin, although infection is hypothetically more likely due to overheating or bacterial contamination of the zygomatic soft tissue. Furthermore, the protrusion of the tip above the zygomatic bone is not a constant in patients with cutaneous fistulae and does not necessarily mean that infection will develop. However, it is bothersome and may require treatment even if it is not infected, as patients usually report discomfort when trying to sleep or apply make-up.

From the literature review [15,17,18,19,20,21,22], few reports have been presented, but the frequency of this complication seems not to be rare or minimal, even if it is not possible to identify a precise value of occurrence. The frequency can vary from 2 to 10% approximatively. Additionally, even in the absence of clear data and comparative studies on the incidence of cutaneous fistula development in the quad zygoma protocol rather than in the unilateral mono zygoma protocol, orbito-zygomatic infection seems to most frequently affect patients treated with two fixators on each side.

When those patients with cutaneous fistula were analysed, some of them showed the same characteristic: insufficient inter-implant space in the apical part of the ZIs [20]. Nevertheless, no authors reported inadequate inter-implant distance between the apical part of the ZIs on the same zygoma as a possible cause of zygomatic fistula and implant failure.

An experimental study [24] investigated the influence of the distance between adjacent implant osteotomies in a polyurethane block on heat accumulation in the interosseous area. When the distance between the centres of the osteotomies is 7 mm, compared to 14 mm, the temperature in the inter-osteotomy area increases and exceeds the threshold for bone thermal necrosis. Heat accumulation in the implant inter-osteotomy area also increases when adjacent drilling has been completed in dense bone [24], as is the case in the zygoma. With the limitations of this study (in vitro study without irrigation), we can assume that in vivo, reducing the distance between osteotomies may also lead to increased temperature in the inter-osteotomy area. In addition, it should be considered that the majority of articles on zygomatic cutaneous fistulae are case reports, which provide little evidence. Despite the limitations of these studies [24], bone resorption is more likely to occur when ZIs are placed too close together. The limited amount of bone available in the zygoma does not easily allow the osteotomies to be spaced far enough apart.

In addition, current surgical techniques do not allow precise placement of ZIs [4,5]. In fact, even guided surgery cannot improve the accuracy of ZI placement compared to freehand surgery due to the extra-long trajectory of ZIs [24,25,26]. This can lead to significant positional errors at the end of the tool trajectory, and the Zis may be placed too close together to avoid the orbit. Furthermore, this effect can be amplified at the level of the outer cortical plate of the zygoma, where the cooling solution barely reaches the tip of the bur. All these factors can lead to bone overheating with a hypothetical risk of bone necrosis and bone resorption with lack of osseointegration and implant failure [24]. Orbito-zygomatic fistulas can result from bone necrosis and infection.

Recent studies have reported, based on computer tomography software, that the amount of malar bone for ZI anchorage is always adequate for the placement of two devices per side. Therefore, if well planned and placed, the placement of two appliances per side can be a reasonable and reliable therapeutic option [25,26]. However, due to the limited number of cases studied (23 edentulous patients), this result may not apply to all zygomas and the amount of zygomatic bone space may not be sufficient in some cases for double monozygomatic implants. Additionally, other adjuvant treatments or unexplored causes have not been fully investigated [27,28,29].

In fact, placing two ZIs monolaterally in the zygomatic bone with optimal distribution is a challenging technique; abundant irrigation must be maintained during drilling and surgeons must maintain a minimum distance of 2 mm between implants, which is critical to avoid thermonecrosis and ensure adequate osseointegration around implants. In addition, it is imperative to maintain a safe distance of 2 mm from the orbital cavity and inferior temporal fossa during planning and surgical procedures [18]. Finally, to achieve better stability, the fixtures could engage both the inner and outer zygomatic cortical plate without protruding.

Given this demanding technical precaution, the unavailability of precise surgical guides and the incidence of peri-zygomatic fistula with possible implant failure, the authors believe that ipsilateral placement of two ZIs should be used with caution. The use of a single ZI combined with a conventional implant in the anterior alveolar crest, possibly preceded by small bone reconstruction (guided bone regeneration or bone graft) in the premaxillary region, should be preferred.

## 4. Conclusions

Despite the use of zygomatic implants in the rehabilitation of the edentulous atrophic maxilla proving to be a predictable procedure, the incidence of complications should not be underestimated. In particular, peri-zygomatic fistula can lead to loss of the implant and prosthetic rehabilitation with a residual scar in the peri-zygomatic area, as in the case presented. From the review of the literature, this complication seems to be not uncommon, even if it is not possible to identify a precise value of the occurrence rate yet. It appears that this extra-oral infection mainly affects patients treated with two implants in the same zygoma. The need for two osteotomies increases the risk of thermal osteonecrosis. The relatively limited amount of zygomatic bone available requires more precise bone drilling and implant placement. Adequate distance from the infra-temporal fossa and orbit must be maintained, and the small amount of residual space may lead to placement of the ZIs too close together. Placement of the ZIs without adequate inter-implant distance, as in the case presented, may lead to bone resorption and infection, with zygomatic cutaneous fistula development and implant failure. Although further research is needed, lack of space between ZI fixtures appears to be one of the main causes of extra-oral ZI infection. Given the increased incidence of extraoral complications with the quad ZIs technique, the placement of two implants in the same zygoma should be limited to certain types of patients when other rehabilitation strategies are no longer possible.

## Data Availability

The original contributions presented in this study are included in the article. Further inquiries can be directed to the corresponding authors.
